# At-home sleep monitoring using generic ear-EEG

**DOI:** 10.3389/fnins.2023.987578

**Published:** 2023-02-01

**Authors:** Yousef R. Tabar, Kaare B. Mikkelsen, Nelly Shenton, Simon L. Kappel, Astrid R. Bertelsen, Reza Nikbakht, Hans O. Toft, Chris H. Henriksen, Martin C. Hemmsen, Mike L. Rank, Marit Otto, Preben Kidmose

**Affiliations:** ^1^Department of Electrical and Computer Engineering, Aarhus University, Aarhus, Denmark; ^2^T&W Engineering A/S, Allerød, Denmark; ^3^Department of Clinical Neurophysiology, Aarhus University Hospital, Aarhus, Denmark

**Keywords:** electroencephalography, sleep monitoring, ear-EEG, long-term sleep monitoring, home recording

## Abstract

**Introduction:**

A device comprising two generic earpieces with embedded dry electrodes for ear-centered electroencephalography (ear-EEG) was developed. The objective was to provide ear-EEG based sleep monitoring to a wide range of the population without tailoring the device to the individual.

**Methods:**

To validate the device ten healthy subjects were recruited for a 12-night sleep study. The study was divided into two parts; part A comprised two nights with both ear-EEG and polysomnography (PSG), and part B comprised 10 nights using only ear-EEG. In addition to the electrophysiological measurements, subjects filled out a questionnaire after each night of sleep.

**Results:**

The subjects reported that the ear-EEG system was easy to use, and that the comfort was better in part B. The performance of the system was validated by comparing automatic sleep scoring based on ear-EEG with PSG-based sleep scoring performed by a professional trained sleep scorer. Cohen’s kappa was used to assess the agreement between the manual and automatic sleep scorings, and the study showed an average kappa value of 0.71. The majority of the 20 recordings from part A yielded a kappa value above 0.7. The study was compared to a companioned study conducted with individualized earpieces. To compare the sleep across the two studies and two parts, 7 different sleeps metrics were calculated based on the automatic sleep scorings. The ear-EEG nights were validated through linear mixed model analysis in which the effects of equipment (individualized vs. generic earpieces), part (PSG and ear-EEG vs. only ear-EEG) and subject were investigated. We found that the subject effect was significant for all computed sleep metrics. Furthermore, the equipment did not show any statistical significant effect on any of the sleep metrics.

**Discussion:**

These results corroborate that generic ear-EEG is a promising alternative to the gold standard PSG for sleep stage monitoring. This will allow sleep stage monitoring to be performed in a less obtrusive way and over longer periods of time, thereby enabling diagnosis and treatment of diseases with associated sleep disorders.

## 1. Introduction

Lack of sleep and poor sleep quality is a grand societal challenge. Poor sleep quality has a negative impact on health, the feeling of wellbeing, quality of life and on human cognitive performance. This has large negative consequences for society, productivity, and economy. Recently, sleep researchers and physicians of sleep medicine have emphasized the importance of sleep on human’s health (Naismith et al., 2011; Galbiati et al., 2019; Stefani and Högl, 2020). However, the development within the field of sleep monitoring has not evolved much beyond today’s gold standard, polysomnography (PSG). As its name describes, PSG is a method that encompasses multiple modalities to describe the sleep. These measurements are typically recorded in sleep clinics and include recordings of brain activity [electroencephalography (EEG)], eye movements [electrooculography (EOG)], muscle [electromyography, (EMG)] and heart activity [electrocardiography (ECG)] (Berry et al., 2012). The PSG’s range of sensors and wiring means that PSG monitoring is discomfortable and in consequence has a negative impact on the sleep. Furthermore, sleep monitoring often takes place in a sleep clinic instead of in the patient’s home environment and sleeping in an unfamiliar environment also has a negative impact on sleep. Although patients may to some extent become accustomed to the equipment and unfamiliar environment, so that the effects become less of a problem over time, the phenomenon is well-known and are called the “first night effect” and implies e.g., a reduced total sleep time, a decrease in sleep efficiency, and delayed and decreased REM sleep (Agnew et al., 1966). These aspects, together with the dependence on health professionals and the significant cost associated has limited the PSG’s use in long-term sleep monitoring. In this light, there is a need for an efficient, comfortable, and easy to use device for monitoring sleep at home.

Alternative sleep assessment methods such as sleep diaries and actigraphy are currently used in many sleep studies and clinical investigations (Sadeh, 2015; Zhu et al., 2018). Unfortunately, the amount of information gained from these methods is limited compared to that of PSG. They are therefore mainly used in parallel with PSG (Gaina et al., 2004). In recent years, several simple monitoring systems have been introduced for sleep assessment. These devices rely on fewer sensors (mostly dry EEG electrodes) to increase comfort and ease of use. The Dreem headband (Arnal et al., 2019), the forehead mounted Prodigy device (Younes et al., 2017) and ear-centered electroencephalography (ear-EEG) device (Mikkelsen et al., 2019; Nakamura et al., 2019) are some examples. While comfort and ease of use make these devices ideal for sleep assessment, they are still to be validated in vaster studies before they can be implemented in the clinic.

Ear-centered electroencephalography was first introduced in 2011 (Looney et al., 2011). The original aim was to provide a more comfortable and affordable solution for several neurophysiological problems at a negligible performance cost. Ear-EEG is a method in which EEG signals are recorded from electrodes in or around the ear. A large variety of different solutions have been proposed, including electrodes placed solely around-the-ear (Bleichner and Debener, 2017), electrodes on customized earpieces (Mikkelsen et al., 2015) and more generic type earpieces (Goverdovsky et al., 2015). One of the most advanced methods are based on dry-contact electrodes embedded on individualized earpieces made of soft silicone (Kappel et al., 2018). The first ear-EEG sleep assessment study was performed in 2019 (Mikkelsen et al., 2019, 2021b; Tabar et al., 2020, 2021) using custom made earplugs and a commercial amplifier. In the current study we deployed a recent advancement in the development of a comfortable, generic, and ready to use setup for sleep assessment, without the sacrifice of performance. In this article, we present an at-home sleep monitoring setup with generic earpieces and a proprietary amplifier for ear-EEG sleep assessment in healthy people.

The focus of this article is on the comfort of the presented ear-EEG device, the data quality of the recordings and the resulting hypnograms. First, we present the generic earpiece design. Then, we introduce our custom EEG amplifier. Next, we present the feedback on the comfort of the earpieces. Finally, the data quality and hypnograms are presented.

## 2. Materials and methods

### 2.1. Experimental setup

#### 2.1.1. The generic earpieces

A prerequisite for this study was the development of a generic earpiece accommodating ear-EEG based sleep monitoring to a wide range of the population. The generic earpiece should provide a reliable and robust contact between the body and the electrodes embedded in the earpiece, and at the same time the earpiece should fit most human ears, be easy to use and be comfortable to sleep with. To begin the design process, we studied the anatomy of the human ear, to identify sizes, curves, and anatomical landmarks. This was achieved by examination of a large number of 3D scans of human ears and through review of existing literature in the domain (Toivonen et al., 2002; Lee et al., 2018; Modabber et al., 2018). This was used as the first input to the design process. The earpiece design process was iterative and in each step in the process we evaluated both the comfort, the ease of use and the EEG signal quality.

There is a very large variety in anatomical shape and sizes of human ears, and to accommodate most ears, it was necessary to design four different earpieces. All four earpieces had the same basic shapes but varied in sizes. The earpieces consisted of three main features: an ear canal part, a tail, and a main body. The ear canal part was given a tulip-like shape to ensure easy insertion in the ear canal, while still filling out the cross section of the ear canal. The ear canal was designed in three different sizes, equivalent to cross sectional diameters of 7, 8, and 9 mm. Essential to the comfort is how deep the earpiece goes into the ear canal and how well it fits the anatomy of the ear. In our design the earpiece was relative shallow and was not going more than 5–6 mm into the ear canal. The tail was designed to keep the earpiece in place by applying pressure on the outer edge of concha. The main body acted as a connector between the two other parts and formed a smooth transition between the concha and the outer part of the ear canal. Additionally, the main body served as a strain relief for the cable to minimize motion artifact from cable pulling. Both the main body and tail were available in two sizes. Eventually we selected four size combinations of the three main features to be used in the study.

The comfort of an earpiece is related to a wide range of factors including mechanical properties of the earpiece material, the number and placement of electrodes, and the ergonomic design. Our experience was that the earpieces should be made of a soft and compliant material. Thus, the earpieces were molded in a soft silicone material (Detax Software 2.0, DETAX GmbH, Germany).

Regarding the number and position of the electrodes, for sleep monitoring the most important factor is to have a good cross-ear derivative; this aspect has been investigated in detail in Mikkelsen et al. (2021a). Thus, the number of electrodes in each ear is a trade-off between comfort and redundancy; increasing the number of electrodes gives a higher degree of redundancy at the cost of lower comfort. In our design we decided to use two recording electrodes in each ear, which is significantly lower than the six electrodes in each ear used in previous studies (Mikkelsen et al., 2017, 2019). The electrodes were placed where the earpiece seemed to apply the largest pressure toward the body, because this is believed to give the best contact between the electrode and the body.

**FIGURE 1:**
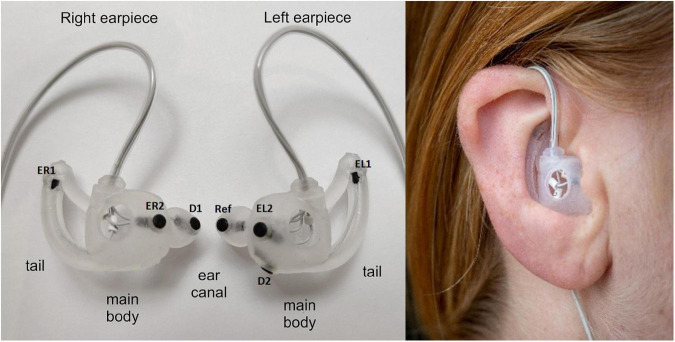
**(Left)** Generic earpieces with mounted electrodes. The placement of the three main features (ear canal, tail, and main body) are specified for each earpiece. The generic earpiece was molded in soft silicone in one piece to ensure good comfort. Cable relief was incorporated in the design to reduce movement artifacts from cable pulling. Also, a formed tube was designed to guide the cables behind the ear to further reduce cable movement, to increase comfort and to make the earpiece more discrete. EL1, EL2, ER1, and Er2: data electrodes, D1 and D2 ground electrodes, ref: reference electrode. **(Right)** Earpiece mounted in the ear.

Finally, the shielded cables where assembled in a form-stable and arc-formed tube guiding the cables around the superior part of the Helix of the ear, see Figure 1. This further reduces the effect of cable movement and make the earpiece more discrete. Electrodes were embedded in the earpieces; the remaining electronics were placed in a box outside the ear to ensure good comfort.

#### 2.1.2. Custom-designed EEG-amplifier and electrodes

The EEG amplifier was a 4-channel “EEG-to-digital converter” application specific integrated circuit (ASIC) specifically developed for ear-EEG measurements. The ASIC was optimized for high input impedance, high common mode rejection, low current noise, and low power consumption. The ASIC was a revised version of the design described in Zhou et al. (2016); it was extended from 2 to 4 channels and a digital control block was included to make it easier to store the data. The EEG amplifier was connected to the electrodes embedded in the earpieces. Two electrodes in each earpiece were used as recording electrodes, one electrode in the left earpiece was used as reference electrode and one electrode in each earpiece was used as ground. The sleep study measurements were all recorded at 250 Hz sampling rate and with 14 bit resolution.

The electrodes were made of Titanium with a porous coating of Iridium Oxide at the contact surface, see (Kappel et al., 2018) for a detailed description and characterization. The electrodes were circular with a diameter of 2.6 mm and with a slight concave shape, whereby the electrodes protruded slightly from the surface of the earpieces. Each electrode was connected to the amplifier with a Ø 0.53 mm coaxial cable. The cable shielding was extended all the way to the back side of the electrode. The shield was actively driven by a unity gain amplifier in the ASIC.

### 2.2. Sleep recordings

This study was approved by the Central Denmark Region Committees on Biomedical Research Ethics (Ref. nr. 1-10-72-13-20) as well as the Danish Medicines Agency (ref. nr. 2020012619). Written informed consent was obtained from the participants prior to participation. 10 subjects (4 f, 6 m) participated in this study. The ages of the subjects ranged between 22 and 35, with a mean of 27.4 years. Participants were screened for hearing loss, sleep disorders, neurological disorders, bruxism, pregnancy, drug usage, allergies, chronic pain, and sleep apnea. Each participant attended an earpiece fitting session prior to the recordings. During this session, the earpieces with the best fit to the participant’s ears was determined by visual inspection of the EEG signal, and the participants were trained in mounting the earpieces themselves. The subjects were instructed to put on the earpieces and start recording whenever they wanted to go to bed and stop the recording when they wake up. They were free to spend any time in bed before sleeping. The participants were asked to fill a sleep diary during the recording and a questionnaire immediately after the wake up.

The study was divided in two parts, Part A and Part B. In Part A, participants slept two nights with the partial PSG (EEG, EOG, and EMG electrodes) and ear-EEG setup. Please refer to Mikkelsen et al. (2019) for more details about the partial PSG. Following two successful recordings in Part A, the study proceeded to Part B in which the participants recorded ten full nights using only the ear-EEG setup. The recordings were performed in the participants’ own home. In Part A, participants visited the laboratory on the day of the recording to get the partial PSG mounted. The participants mounted the earpieces themselves just before the start of the recording. Ear-EEG and partial PSG were recorded using different data acquisition devices. The participants were asked to press a trigger button on both devices simultaneously for signal alignment. The partial PSG recordings were manually scored by a trained professional sleep technologist according to the AASM manual for the Scoring of Sleep and Associated Events (Berry et al., 2012).

**FIGURE 2:**
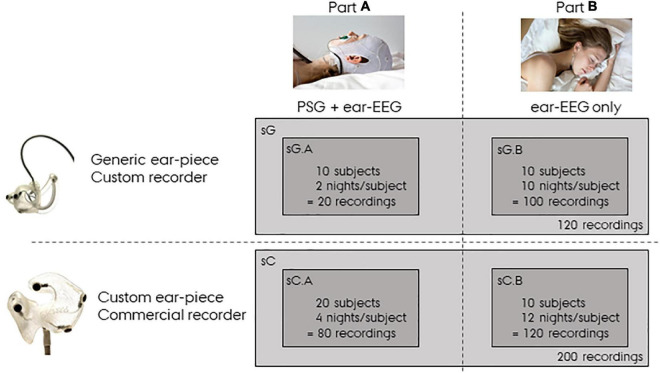
Overview of the data. sG refers to data from the study with *generic* earpieces, and sC to data from the study with *custom* earpieces. Both datasets have a Part A and a Part B. Part A were recorded with both polysomnography (PSG) and ear-centered electroencephalography (ear-EEG), whereas Part B were recorded from ear-EEG only.

In the following sections the recordings described above will be referred to as sG, a dense form referring to the study with generic earpieces. Specifically, sG will refer to the complete dataset, and sG.A and sG.B will refer to the dataset related to Part A and Part B, respectively. In addition to this dataset, we also used a companioned dataset from a previous ear-EEG sleep monitoring study (Mikkelsen et al., 2019, 2022) in which custom-made earpieces and a commercial amplifier were used. This dataset will be referred to as sC, a dense form referring to the study with custom earpieces. sC was structured in the same way as sG, but with 20 subjects recorded four nights with both partial PSG and ear-EEG in part A (referred to as sC.A) and 10 subjects recorded 12 nights with only ear-EEG in part B (referred to as sC.B). A summary of the datasets used in this article is presented in Figure 2. It is important to note that in the current study, sC was only used for training the sleep scoring classifier and for statistical comparisons.

### 2.3. Assessment of sleep quality, comfort and ease-of-use

**FIGURE 3:**
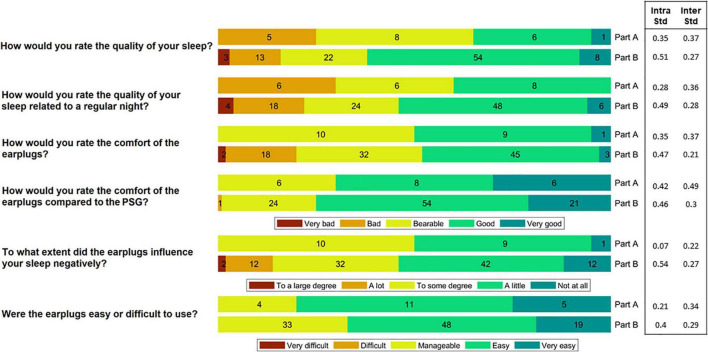
The participants rated the comfort and ease-of-use of the device after each night. In Part A, they slept wearing both the polysomnography (PSG) setup and the earpieces, whereas in Part B they only wore the earpieces. This figure shows a summary of their ratings. The numbers in the bars reflect the number of responses (20 in total for Part A and 100 in total for Part B). Inter subject variation (Inter Std) and Intra subject variation (Intra Std) are shown for each question-part combination.

The participants reported perceived sleep quality, comfort and ease-of-use of the device by rating six questions on a Likert scale. The six questions and the corresponding Likert scale can be seen in Figure 3. The participants were instructed to fill out the questionnaire just after they woke up in the morning. Inter, intra subject variation values were computed as the standard deviation of the ratings between the subjects and within the subjects.

### 2.4. Signal pre-processing and channel selection

A signal pre-processing pipeline was applied to the recorded ear-EEG data. Each channel was bandpass filtered (0.1–100 Hz), and notch filtered at 50 and 100 Hz to suppress power line noise. Artifacts were identified and removed following several steps. For the sG dataset we observed periodic noise spikes with a period of 200 ms. The noise was related to internal communication in custom developed amplifier and the severity of the noise increased with electrode impedance, i.e., the higher the electrode impedance the more prone the channel was to the device induced noise. The noise was detected by an algorithm looking for spikes with a repetition rate of 200 ms. If a spike was detected it was removed and the signal was interpolated based on the clean signal in the neighboring samples.

Spikes of short duration and high amplitude were also detected and removed. These spikes are usually due to small changes in the electrode-skin connection. Poor electrode-skin connection can also lead to dominant high frequency noise in the signal. Long periods (>30 s) with such a noise were detected by thresholding the high frequency power. Finally, any sample with an absolute value greater than 350 μV were rejected. Exclusion of unsuccessful recordings from the dataset was performed if more than 30% of the signal was noisy or if the duration of the recording was less than 5 h.

In the Supplementary material, we have supplied figures showing the effect of both spike removal and general noise removal.

Finally, the four data channels were combined to construct a single channel signal. To extract this signal, a channel selection method based on root mean square (RMS) was employed. The method relied on the idea that noisy signals tend to yield higher RMS values. Accordingly, for every 30 s epoch, RMS values were computed for any possible cross-ear combination of the channels. The combinations were constructed by using channels from right ear [ER1, ER2, and Avg(ER1,ER2)] referenced to the channels from left ear [EL1, EL2, Avg(EL1,EL2), and ref]. The channel combination that yielded that lowest RMS value was selected.

### 2.5. Automatic sleep scoring

Following the pre-processing step and the construction of a single channel signal, an automatic sleep scoring algorithm was applied to the resulting signal. The goal of the automatic sleep scoring was to assign a correct sleep stage (N1, N2, N3, REM, and wake) to every 30 s epoch.

First, the signal was segmented into 30 s long epochs. Then, a feature extraction step was applied to every epoch resulting in 84 features for each epoch. The feature set was selected to include time domain, frequency domain, Continuous Wavelet Transform (CWT) based, EMG proxy, EOG proxy, sleep event proxies, and non-linear features [adapted from Mikkelsen et al. (2019)]. The list of features is presented in Supplementary Table 1. Since sC and sG were recorded with different devices, the computed features were normalized using the normalize function in MATLAB. The normalization process was applied to each recording separately.

The epochs were then classified using a five-class random forest classifier consisting of 100 decision trees. This method has been used in several ear-EEG sleep scoring studies (Khademi et al., 2018; Mikkelsen et al., 2019; Tabar et al., 2021). The classification performance was measured using Cohen’s kappa value (Cohen, 1960) which reflects the agreement between automatic and manual scoring. A “leave-one-subject-out” (LOSO) cross-validation was used to validate the results. This means that for each subject, the classifier was trained using only recordings from the remaining subjects. Since manual scoring was only available for the Part A recordings, the classification results were computed only for Part A.

### 2.6. Analysis of the effects of equipment, part, and subject

The Part B recordings were performed without PSG and therefore the performance of the automatic sleep scoring cannot be validated using Cohen’s kappa value. Instead, we computed the following sleep metrics for all recordings:


(1) $$R\,E\,M\,f\,r=\frac{n\,u\,m\,b\,e\,r\,o\,f\,R\,E\,M\,e\,p\,o\,c\,h\,s}{t\,o\,t\,a\,l\,n\,u\,m\,b\,e\,r\,o\,f\,e\,p\,o\,c\,h\,s}$$

(2) $$N\,3\,f\,r=\frac{n\,u\,m\,b\,e\,r\,o\,f\,N\,3\,e\,p\,o\,c\,h\,s}{t\,o\,t\,a\,l\,n\,u\,m\,b\,e\,r\,o\,f\,e\,p\,o\,c\,h\,s}$$

(3) $$S\,E\,\left(s\,l\,e\,e\,p\,e\,f\,f\,i\,c\,i\,e\,n\,c\,y\right)=\frac{s\,l\,e\,e\,p\,d\,u\,r\,a\,t\,i\,o\,n}{d\,u\,r\,a\,t\,i\,o\,n\,o\,f\,t\,h\,e\,r\,e\,c\,o\,r\,d\,i\,n\,g\,a\,f\,t\,e\,r\,f\,i\,r\,s\,t\,s\,l\,e\,e\,p\,e\,p\,o\,c\,h}$$

(4) $$N\,R\,E\,M\,t\,o\,N\,R\,E\,M=\frac{n\,u\,m\,b\,e\,r\,o\,f\,N\,R\,E\,M\,t\,o\,N\,R\,E\,M\,t\,r\,a\,n\,s\,i\,t\,i\,o\,n\,s}{t\,o\,t\,a\,l\,n\,u\,m\,b\,e\,r\,o\,f\,N\,R\,E\,M\,e\,p\,o\,c\,h\,s}$$

(5) $$N\,R\,E\,M\,t\,o\,R\,E\,M=\frac{n\,u\,m\,b\,e\,r\,o\,f\,N\,R\,E\,M\,t\,o\,R\,E\,M\,t\,r\,a\,n\,s\,i\,t\,i\,o\,n\,s}{t\,o\,t\,a\,l\,n\,u\,m\,b\,e\,r\,o\,f\,N\,R\,E\,M\,e\,p\,o\,c\,h\,s}$$

(6) $$R\,E\,M\,t\,o\,R\,E\,M=\frac{n\,u\,m\,b\,e\,r\,o\,f\,R\,E\,M\,t\,o\,R\,E\,M\,t\,r\,a\,n\,s\,i\,t\,i\,o\,n\,s}{t\,o\,t\,a\,l\,n\,u\,m\,b\,e\,r\,o\,f\,R\,E\,M\,e\,p\,o\,c\,h\,s}$$

(7) $$R\,E\,M\,t\,o\,N\,R\,E\,M=\frac{n\,u\,m\,b\,e\,r\,o\,f\,R\,E\,M\,t\,o\,N\,R\,E\,M\,t\,r\,a\,n\,s\,i\,t\,i\,o\,n\,s}{t\,o\,t\,a\,l\,n\,u\,m\,b\,e\,r\,o\,f\,R\,E\,M\,e\,p\,o\,c\,h\,s}$$

We investigated the effect of equipment (individualized vs. generic earpieces), part (PSG and ear-EEG vs. only ear-EEG) and subject on each of these metrics using Linear Mixed Models (LMM). The objective was to investigate if differences in sleep characteristics could be explained by the equipment, part or inter-subject variation. For each sleep metric, a LMM was designed as [in Wilkinson notation (Wilkinson and Rogers, 1973)]:


(8) Sleepmetric∼1+subject+part+study+(1|recording)

Where equipment = 1, 2 (corresponding to individualized and generic earpieces), part = A, B (corresponding to combined PSG and ear-EEG, and only ear-EEG), and subject = 1, …, 20 were included as fixed effects and recording = 1, …, 16 / recording = 1, …, 12 was included in the model as a random effect. By fitting the models to the data, we examined the effect of each variable on the sleep metrics.

## 3. Results

Ten healthy participants (6 m/4 f) aged 27.4 ± 4.9 years were included in the study. From these 10 subjects, we collected 20 nights of combined PSG and ear-EEG and 100 nights of ear-EEG sleep recordings. Three recordings from Part A and nine recordings from Part B were rejected and repeated due to ear-EEG recording problems. The reasons for rejection of these recordings were: bad earpiece mounting (five recordings), low battery on recording device (four recordings) and earpiece fallen out of the ear during sleep (three recordings). Partial PSG recordings were not checked for noise. However, two recordings were repeated due to failure of the acquisition device.

### 3.1. Comfort and ease-of-use

Following each night, the participants rated the comfort and ease-of-use of the device by answering a questionnaire. A summary of the answers is illustrated in Figure 3. During the part of the study where sleep was assessed using both PSG and ear-EEG (Part A), 65% of the responses reported that sleep quality was “bad” or “bearable.” In part B, using only the generic earpiece, 62% reported a “good” or “very good” sleep quality. This shift in perceived sleep quality fits well with their ratings of the comfort of the earplugs compared to the PSG setup, where 75% of the responses showed that the comfort was “good” or “very good.” Inter and intra subject variation values are also presented in Figure 3. These values were observed to be generally low for all question-part combinations.

### 3.2. Data quality

**FIGURE 4:**
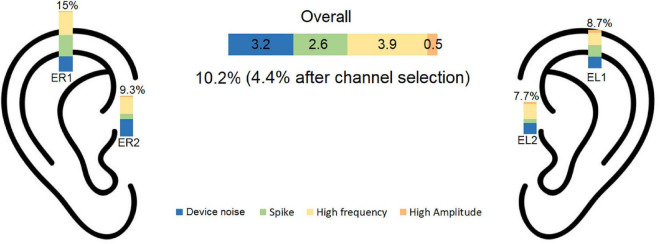
Percentage of rejected data is illustrated for each noise category for each electrode. The overall rejection percentage was 10.2% before and 4.4% after channel selection. EL1, left electrode 1; EL2, left electrode 2; ER1, right electrode 1; ER2, right electrode 2.

The artifact rejection procedure described in section “2.4. Signal pre-processing and channel selection” led to rejection of on average 10.2% of the data. The proportion of each artifact rejection criterion was: device related noise: 3.2%, spikes 2.6%, high frequency: 3.9%, high amplitude: 0.5%. A summary of the pre-processing data rejection is presented in Figure 4.

The channel selection procedure described in section “2.4. Signal pre-processing and channel selection” was used to find the cross-ear combination with the least RMS among the accepted channels. Using this procedure, the average number of rejected epochs was 4.4%. The inter subject variation for data rejection was 0.027 for part A and 0.036 for part B.

### 3.3. Sleep scoring algorithm

**FIGURE 5:**
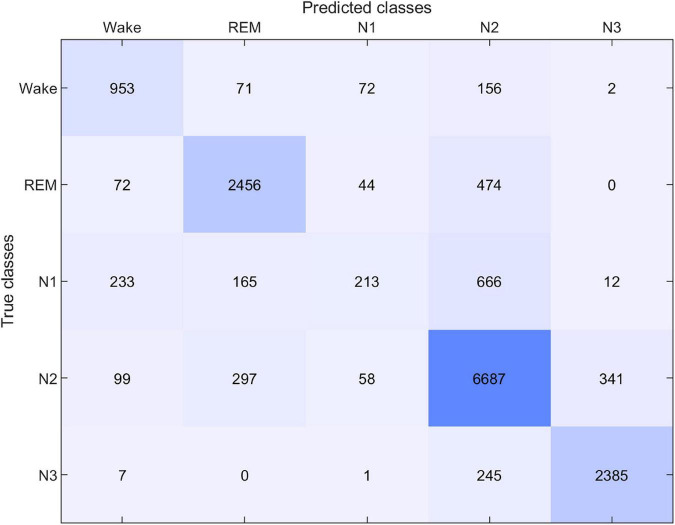
Confusion matrix for sleep scoring with the sCGsG cross-validation method.

The performance of the sleep scoring algorithm was validated using the sC.A and sG.A datasets. Different training and testing strategies were used to assess the sleep scoring, in which LOSO cross validation was used where applicable. sC.A included 80 recordings from 20 participants and sG.A included 20 recordings from 10 participants. The classifier was trained separately using sC.A, sG.A and a combination of sC.A and sG.A, and tested on both sC.A and sG.A. For simplicity, we called each of these cross-validation schemes XY, where X is the train set and Y is the test set, e.g., sCGsG means trained with sC.A and sG.A combined and tested on sG.A. A summary of the results of the different combinations is shown in Table 1. The confusion matrix for 5 class classification using the sCGsG method is presented in Figure 5.

**TABLE 1:** The kappa values for each train-test pair together with the applied cross validation method.

Method	sCsC	sCsG	sGsG	sGsC	sCGsC	sCGsG
Training set	sC.A	sC.A	sG.A	sG.A	sC.A,sG.A	sC.A,sG.A
Test set	sC.A	sG.A	sG.A	sC.A	sC.A	sG.A
Cross validation	LOSO	–	LOSO	–	LOSO	LOSO
kappa	0.73	0.68	0.69	0.66	0.73	**0.71**

Highest performance on the sG.A dataset is marked in bold.

While the average kappa value for sCsG was 0.68, it increased to 0.69 for the sGsG method. The number of recordings in the training set was only 18 in sGsG compared to 80 in sCsG. However, it still resulted in slightly better classification performance. The average kappa value further increased to 0.71 when both datasets were included in the training set in sCGsG. This is the highest kappa value we achieved for sG.A.

**FIGURE 6:**
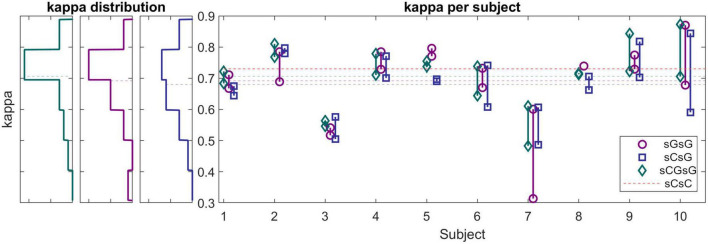
**(Left panels)** Histograms of kappa values for sG recordings (cross-validation scheme: sGsG: purple, sCsG: blue, sCGsG: green). Dashed lines represent the average of each method. For the sGsG and sCGsG schemes, the majority of the recordings yielded a kappa value above 0.7. **(Right panel)** Kappa values for each subject for all three cross-validation schemes. Each point represents one recording.

The right panel in Figure 6 shows the kappa values for each recording using the sCsG, sGsG and sCsG cross validation scheme. The kappa values of subjects 3 and 7 were conspicuously lower than for the other subjects. The scoring of the remaining 7 subjects resulted in a mean kappa well over 0.7 regardless of the training set. The three left panels in Figure 6 shows the distribution of kappa values for each cross-validation scheme. For the sGsG and sCGsG cross validation schemes the majority of the recordings have kappa values above 0.7. The average kappa value is shown for each method with a colored dashed line.

**FIGURE 7:**
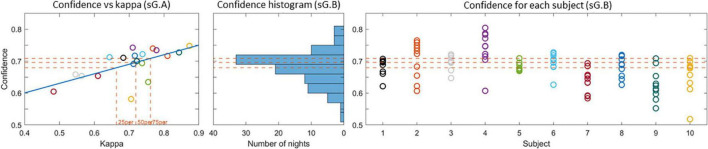
**(Left)** Confidence versus kappa value for the sG.A recordings. The 25th, 50th, and 75th percentiles of the kappa values and the corresponding confidences are shown with dashed lines. **(Center)** Histogram of the confidence values for the sG.B recordings. **(Right)** Confidence values for each recording. The 25th, 50th, and 75th percentile confidence values are presented with dashed lines in all plots.

The proportion of decision trees voting for a given sleep stage can be interpreted as an estimate of the likelihood for that sleep stage. The output of the classifier is the sleep stage with the largest proportion of votes, and the proportion itself can be interpreted as a confidence measure of the classifier’s decision. In a previous study (Mikkelsen et al., 2020), we observed that the mean value of the confidence measure across all epochs in a recording is a reliable estimate of the overall scoring performance. Thereby the confidence measure can be used to assess the sleep scoring performance for unlabeled data. To illustrate this relation between kappa and confidence the left panel in Figure 7 shows the confidence versus the kappa for the sG.A recordings. It is clear that the confidence values correlate positively with kappa values, which corroborate that the confidence is a reliable estimate of the kappa value. For these G.A recordings, the 25th, 50th, and 75th percentiles of the kappa values were 0.66, 0.72, and 0.76, respectively. In other words 75% of the recordings had a kappa value larger than 0.66, and the 25% best recordings had a kappa value larger than 0.76. The corresponding confidence values were 0.68, 0.70, and 0.71, respectively. The distribution of the confidence values for the sG.B recordings are shown in the second and third subfigure of Figure 7. In 58% of the Part B recordings, a confidence value above 25th percentile of Part A was achieved. This value was 42% for 50th percentile and 26% for 75th percentile. All subjects had at least 2 recordings with a confidence above the 25th percentile.

The average kappa value for the sCsC method was found to be 0.73 which is similar to the value reported in Mikkelsen et al. (2019).

### 3.4. Effect of equipment, part and subject

**FIGURE 8:**
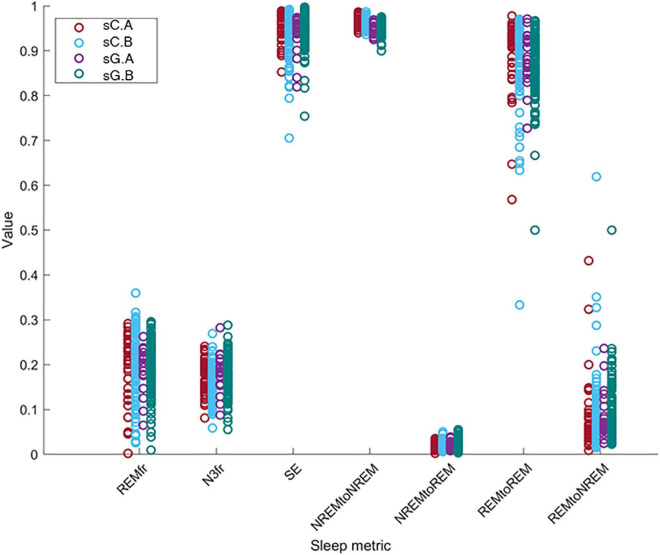
The distribution of the value of the sleep metrics (REMfr, N3fr, SE, NREM to NREM, NREM to REM, REM to REM, and REM to NREM) for sC.A, sC.B, sG.A, and sG.B datasets show a large overlap and no clear equipment or part differences.

We investigated the change of the different sleep metrics with the equipment, part and subject differences. In order to compare the computed sleep metric values between different sets, these values is shown in Figure 8. The distributions of these metrics for sC.A, sC.B, sG.A, and sG.B are presented with different colors. Each circle indicates the value of the related sleep metric for one subject. We observed similar distributions in the data for the different studies and parts. It should be noted that the number of recordings in Part B is considerably larger than in Part A for both studies.

The results of the LMM analysis are presented in Table 2. Each of the models in section “2.5. Automatic sleep scoring” were applied to the sC, sG and sCsG datasets separately. The *p*-value was computed for each fixed effect (subject, part, and equipment).

**TABLE 2:** Results from the linear mixed model (LMM) analysis on the sleep metrics.

Metric	Effect	sC	sG	sCsG
REMfr	Subject	*P* < 0.01*	*P* < 0.01*	*P* < 0.01*
	Part	*P* = 0.16	*P* = 0.87	*P* = 0.26
	Equipment	NA	NA	*P* = 0.83
N3fr	Subject	*P* < 0.01*	*P* < 0.01*	*P* < 0.01*
	Part	*P* = 0.18	*P* = 0.38	*P* < 0.01*
	Equipment	NA	NA	*P* = 0.46
SE	Subject	*P* < 0.01*	*P* = 0.02*	*P* < 0.01*
	Part	*P* = 0.17	*P* = 0.09	*P* = 0.92
	Equipment	NA	NA	*P* = 0.74
NREM to NREM	Subject	*P* < 0.01 *	*P* < 0.01*	*P* < 0.01*
	Part	*P* = 0.30	*P* = 0.18	*P* = 0.69
	Equipment	NA	NA	*P* = 0.13
NREM to REM	Subject	*P* < 0.01*	*P* < 0.01*	*P* < 0.01*
	Part	*P* = 0.31	*P* = 0.31	*P* = 0.20
	Equipment	NA	NA	*P* = 0.16
REM to REM	Subject	*P* < 0.01*	*P* < 0.01*	*P* < 0.01*
	Part	*P* = 0.28	*P* = 0.43	*P* = 0.49
	Equipment	NA	NA	*P* = 0.43
REM to NREM	Subject	*P* < 0.01*	*P* < 0.01*	*P* < 0.01*
	Part	*P* = 0.11	*P* = 0.39	*P* = 0.23
	Equipment	NA	NA	*P* = 0.49

The subject effect was significant for all sleep metrics. The part effect was only significant in the combined dataset in the fraction of N3 sleep. The equipment effect was insignificant for all sleep metrics.

*Significant effects are marked.

The Subject parameter had a significant effect on the variation of all metrics for all datasets. Furthermore, the Part effect was only significantly different in the N3fr metric for sCsG dataset. The model coefficient for the fraction of N3 sleep (N3fr) in the combined dataset sCsG was 2.8%. The Part effect was insignificant in all other metric/dataset combinations. The Equipment parameter was also insignificant for all the metrics and datasets.

## 4. Discussion

This study evaluated a new generic ear-EEG system for sleep monitoring. The system comprised a set of generic earpieces designed to fit most human ears, and the earpieces were connected to a recording device comprising a custom 4-channel “EEG-to-digital converter” ASIC. Both the EEG signal quality and the comfort are essential for a good sleep monitoring device, and the design of the earpieces are important for both these key parameters. The signal quality is intimately related to the electrode-skin interface, and the purpose of the earpiece is to provide a firm and stable electrode-skin connection. The comfort of an earpiece is related to a wide range of factors including mechanical properties of the earpiece material, the number and placement of the electrodes embedded in the earpiece, and the ergonomic design. The earpiece design process was iterative and in each step in the process we evaluated both the comfort and the signal quality. Some of the key experiences obtained in the process, and the consequences for the earpiece design, are summarized here: The earpieces need to be made of a soft and compliant material; in our design the earpieces were made of a silicone material with shore 60. Essential to the ergonomic design is how deep the earpiece goes into the ear canal and how well it fits the anatomy of the ear. In our design the earpiece was relative shallow and was not going more than 5–6 mm into the ear canal.

The signal quality was quantified in terms of the percentage of rejected data epochs after the pre-processing step. Rejection of epochs with poor signal quality is a common practice in sleep monitoring. Poor signal quality can be due to several factors, but the signal quality depends in almost all situations on the electrode-skin contact. Thus, if the signal quality is challenged by e.g., movements, the effect on the signal quality will be further exacerbated by a poor electrode-skin connection. Therefore, the proportion of rejected epochs is a good measure for assessing the quality of the electrode-skin connection–the lower the proportion of rejected epochs the better the electrode-skin interface. In the companion study (sC), using individualized earpieces with six electrodes in each ear and a commercial EEG amplifier, 9% of the epochs were rejected (Mikkelsen et al., 2019). The amount of rejected epochs after the single channel signal construction was 4.3%. In this study (sG), we recorded 20 nights with partial PSG and ear-EEG, as well as 100 nights with only ear-EEG. In these 120 nights of recording, the pre-processing pipeline rejected 10.2% of the epochs, which decreased to 4.4% after single channel signal construction. This value is very similar to our previous rejection rate. This demonstrates that changing from custom made to generic earpieces and lowering the number of electrodes did not have a significant effect on the cross-ear single channel signal’s quality.

During the study, we identified several factors that caused poor signal quality and occasional rejection of the recording. The main factor was fitting the earpieces. We observed that the subjects were able to easily fit the earpieces after the third or fourth night. Although, the subjects were able to fit the earpieces properly, in some cases, the subject relocated the earpiece during the night without properly fitting it causing the electrodes to lose the connection. In one other case, the subject had excessive movement during the night, which caused the earpiece to fall out of the ear. Otherwise, the subjects were successful in fitting the earpieces. In general, subjects were able to use the device without any problem. Only in few cases, the subjects forgot to charge the device before the recording. We believe that the device can be used easily by a normal user.

Comfort and unobtrusiveness are paramount in long-term sleep recording for at least two reasons: (1) If the sleep monitoring device is not sufficiently comfortable, subjects will not endure it and thus the disadvantages in terms of discomfort will outweigh the benefits of the sleep monitoring. (2) If the monitoring device interferes with the sleep, the sleep information acquired will be biased and thus not provide an accurate impression of the sleep. Therefore, subjects were asked to answer questionnaires after every night to rate their perceived sleep quality, their perceived comfort, the ease of use of the device and whether their sleep had been affected by the earpiece. Based on their answers, we saw that the comfort of the generic earpieces was similar to that of custom-made earpieces from our previous study (Mikkelsen et al., 2019). One of the proposed reasons for this is that it was previously discovered that the depth of the ear canal part of the earpiece was highly correlated with the perceived comfort of the earplug. The generic earpiece was designed to not go further than the second bend of the ear canal, which is the same depth as what the custom earplugs are modeled to. This could explain why the comfort assessments are similar to our previous study. Another important feature in the development of a generic ear-EEG system is the ease-of-use, when asked, most of the subjects reported that the earpieces were easy to mount. In general, subjects preferred the earplugs to the partial PSG setup. The participants responded to comfort and sleep quality questions with a few bad/very bad answers. For most of the questions, the intra subject variation was observed to be higher than the inter subject variation. This shows that the comfort changes more with the recording night rather than the subject.

Another important aspect to consider in this new approach to sleep monitoring is its performance in sleep analysis and scoring. The first part of it, which was mentioned earlier, is the data quality. Poor data quality and missing data is detrimental for sleep scoring. Fortunately, the new earpiece design and the introduction of the customized and application specific amplifier and recording device, did not affect the amount of data rejection. Next, we evaluated the performance of our sleep scoring algorithm on different combinations of this current dataset (sG.A) and a dataset (sC.A) collected in a previous study, with custom earpieces and a commercial amplifier. By combining the datasets, we augment our database and thereby the training and test set of our automatic sleep scoring algorithm. Although the earpiece, amplifier and the number of electrodes were different in those studies, we were able to perform successful automatic sleep scoring by training with one dataset and testing on the other one. First, we observed that training and testing on sG.A yielded a higher performance than training on sC.A and testing on sG.A. This is probably reflecting a combination of two effects: (i) the new setup introduces a certain unique fingerprint in the recordings which is not explained by sC.A. (ii) The characteristic features of sleep in the ear-EEG are largely preserved across the two datasets. Combining the sC.A and sG.A datasets in the training set increased the scoring performance for the sG.A dataset. This combination probably allowed the algorithm to generalize over a larger population of subjects and still learn the unique fingerprints of the new setup. However, combining the datasets did not increase the performance on the old dataset (sC.A). This is likely because sC.A is a sufficiently large dataset for the algorithm to generalize over the population of subjects, therefore adding more training data from sG.A does not add any significant new information. Ultimately, the algorithm continues to have most of its knowledge from sC.A and therefore no increase is observed in the performance on sC.A. The performance of the sCGsG scheme was lower than for the sCGsC scheme. It should be mentioned that sC.A is 4 times larger than sG.A, wherefore train-test on sC.A gives a better generalization. We expect this difference to diminish significantly as our database grows. Subject-wise, we observed that two subjects had considerably lower kappa values compared to the other subjects in all dataset combinations. We suggest that this is caused by different subject specific factors like earplug fitting, sleep characteristics and sleep environment. According to Figure 5, the sCGsG method was successful in detecting N2 and N3 stages and less successful in detecting N1 stage. This is similar to what we observed in our previous studies (Mikkelsen et al., 2019; Tabar et al., 2021).

One important aspect of the proposed sleep monitoring system is to provide longitudinal sleep recordings via only ear-EEG recordings. Therefore, it is critical to predict the performance of the sleep scoring algorithm in the part B recordings. While the lack of manual scorings in part B makes it impossible to validate the automatic scorings in the conventional way, we validate them using two analytical methods. The first method is based on the confidence measure derived from the random forest classifier. In a previous study (Mikkelsen et al., 2020), we found a strong correlation between the confidence measure and the kappa value, and this strong relationship was also observed in the current study. This allows us to predict the expected kappa value for part B recordings based on their confidence values. While there is not a one-to-one correspondence between the confidence measure and the kappa value it still provides a good assessment of the performance for the unlabelled data in Part B. As a result, we expect 42% of the Part B recordings to have kappa value above 0.72 which is the median kappa value for part A. Additionally, 58% of the part B recordings were predicted to have kappa value over 0.66 (25th percentile), where 26% of them were predicted to have kappa over 0.76 (75th percentile). While the distribution of the predicted kappa values was highly subject dependent, all the recordings had at least 2 recordings with a kappa above 0.66.

In the second method, we investigated the sleep characteristics of the recordings to see if there was a difference between the estimated sleep characteristics of part A and B for any individual subject. We also aimed to explore whether the new devices (study effect) changed the distribution of the sleep characteristics and ultimately the sleep of subjects. Linear mixed models were constructed for six sleep metrics in which the effects of equipment, part and subject on the sleep metric were investigated. The part effect reflected the effect of the partial PSG setup on the sleep characteristics. We found that the effect of the partial PSG setup was only significant in one sleep metric, namely the fraction NREM stage 3 (N3) sleep for the sCsG dataset. In this case the fraction of N3 sleep increased by 2.8 % from Part A (partial PSG + ear-EEG) to Part B (ear-EEG). The effect of part on all other sleep metrics were insignificant.

Furthermore, we did not find any significant effect of the equipment on any of the sleep metrics, which means that the effect of the new generic earpiece with two electrodes and the new amplifier is negligible. This is in concordance with the fact that we were able to get high classification performance by combining both datasets. The subject effect was significant for all sleep metrics. This shows that changes in the sleep characteristics were related to subject specific idiosyncrasies rather than from which part or which equipment the recordings came from.

These results suggests that generic ear-EEG is a promising method for long-term sleep monitoring. This may have application within e.g., treatment of diseases such as insomnia, chronic pain and many psychiatric disorders, prognostication of recovery after stroke, concussion and traumatic brain injury, and as an early biomarker in neurodegenerative diseases such as Parkinson’s disease, Levy Body dementia and Alzheimer’s disease.

## 5. Conclusion

A comfortable and unobtrusive device for long-term sleep monitoring will have great clinical value in diagnosis and treatment of many diseases. We have developed an ear-EEG based sleep monitoring device based on a generic ear-EEG with two recording electrodes in each ear, a proprietary amplifier and an associated automatic sleep scoring algorithm. In this study, we have assessed perceived sleep quality, comfort and ease-of-use and compared the sleep scoring performance against partial PSG scoring. The proposed generic earpiece design was found to be as comfortable as the custom-made design. The EEG signal was recorded from 2 electrodes in each ear with an amplifier made specifically for the current application. The quality of the recorded signal was similar to our previous setup, resulting in successful sleep scoring with an average kappa value equal to 0.71. Automatic sleep scoring was also applied to recordings where no manual scoring was available. We used a confidence measure provided by the sleep stage classifier for assessing the quality of the unlabelled sleep scorings and found that 42% of the part B recordings were estimated to have kappa value above 0.72. Finally, we analyzed the sleep patterns, based on a linear mixed model analysis of seven different sleep metrics, and did not find any statistically significant differences between individualized and generic earpieces, or between PSG-nights and ear-EEG nights. These results suggests that sleep monitoring based on generic ear-EEG devices is a promising alternative to PSG for long-term monitoring of sleep stages.

## Data availability statement

The datasets presented in this article are not readily available because the Danish Medicines Agency requires traceability of data in 5 years after completion of the study. To comply with regulations, the data cannot be shared without anonymization of all copies of the dataset. Therefore, data cannot be shared before at earliest at the end of the 5 years period. Until then, the authors are happy to share aggregated statistics presented in this article. Requests to access the datasets should be directed to the corresponding author.

## Ethics statement

The studies involving human participants were reviewed and approved by the Central Denmark Region Committees on Biomedical Research Ethics (case no. 1-10-72-13-20). The patients/participants provided their written informed consent to participate in this study.

## Author contributions

YT: design of experiment, data collection, ear-EEG data analysis, and manuscript writing. KM: conception of study, design of experiment, and ear-EEG data analysis. NS: analysis of questionnaire data and manuscript writing. SK: experimental setup, design and validation of ear-EEG and PSG-platform. AB, RN, HT, and CH: design and validation of generic ear-EEG earpieces. MH: design of experiment and input to data analysis pipeline. MR: conception of study, design of experiment, and input to data analysis pipeline. MO: supervised PSG scorings and clinical guidance. PK: conception of study, design of experiment, design of ear-EEG platform, data analysis, and manuscript writing. All authors approved the final version of the manuscript and agreed to be accountable for all aspects of the work in ensuring that questions related to the integrity of any part of the work have been appropriately investigated and resolved.
