# Supplementary material for: At-home sleep monitoring using generic ear-EEG
Source: Front Neurosci. 2023 Feb 1;17:987578. doi: 10.3389/fnins.2023.987578 (PMC9928964; doi:10.3389/fnins.2023.987578)

**Supplementary Material**

**Supplementary table *.** List of the features used for ear-EEG classification

| # | **Feature name** | **Group** |
| --- | --- | --- |
| 1 | Signal Skewness | EEG Time Domain |
| 2 | Signal Kurtosis |  |
| 3 | Zero Crossing Rate |  |
| 4 | Hjorth Mobility |  |
| 5 | Hjorth Complexity |  |
| 6 | 75th Percentile |  |
| 7 | Channel Correlation |  |
| 8 | Power [50 100 ] Hz band | EMG proxy |
| 9 | Minimal [50 100 ] Hz band Power |  |
| 10 | Relative [50 100 ] Hz band burst Amplitude |  |
| 11 | Slow Eye Movement Power | EOG proxy |
| 12 | Rapid Eye Movement power |  |
| 13-16 | Relative power in α, β, θ, δ bands | EEG Frequency Domain |
| 17-23 | Power-ratios: δ/θ, θ/α, α/β, β/γ, (θ + δ)/(α + β) |  |
| 24 | Spectral edge frequency |  |
| 25 | Median power frequency |  |
| 26 | Mean spectral edge frequency difference |  |
| 27 | Peak power Frequency |  |
| 28 | Spectral Entropy |  |
| 29 | Spindle probability | Sleep event proxies |
| 30 | Frequency stationarity |  |
| 31 | Lowest adj. frequency similarity |  |
| 32 | Maximum B-spline transform |  |
| 33 | Longest sleep spindle |  |
| 34-39 | Power mean in *lf,* γ, α, β, θ, δ bands | CWT based features |
| 40-45 | Power variance in *lf,* γ, α, β, θ, δ bands |  |
| 46-51 | Duration of the activation in *lf,* γ, α, β, θ, δ bands |  |
| 52-57 | Maximum power in *lf,* γ, α, β, θ, δ bands |  |
| 58-63 | Relative power in *lf,* γ, α, β, θ, δ bands |  |
| 64-78 | Power ratios: δ/θ, θ/α, α/β, β/γ |  |
| 79:84 | MSE1-5,LZC | Nonlinear features |

# Noise removal examples

In the two figures below, we demonstrate the effects of the noise removal pipeline, as described in Section 2.4 in the manuscript. The first figure, we see a train of 4 spikes with a 200 ms interval, which have been detected and removed based on the surrounding EEG. In the second plot, a roughly 50 second period of movement artifacts have been detected and removed.


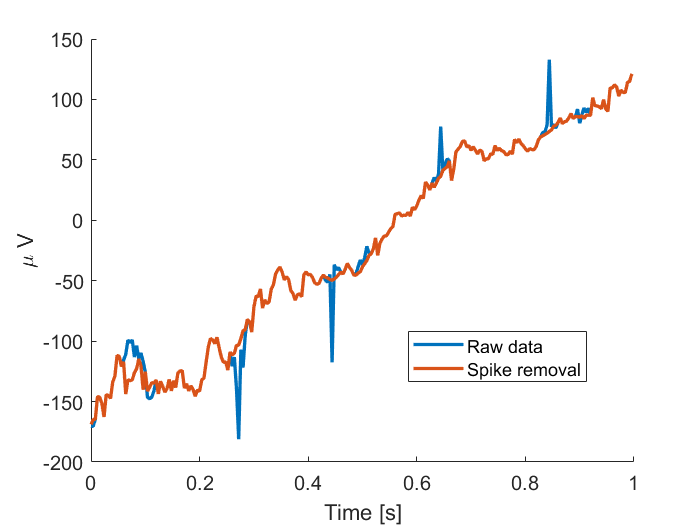


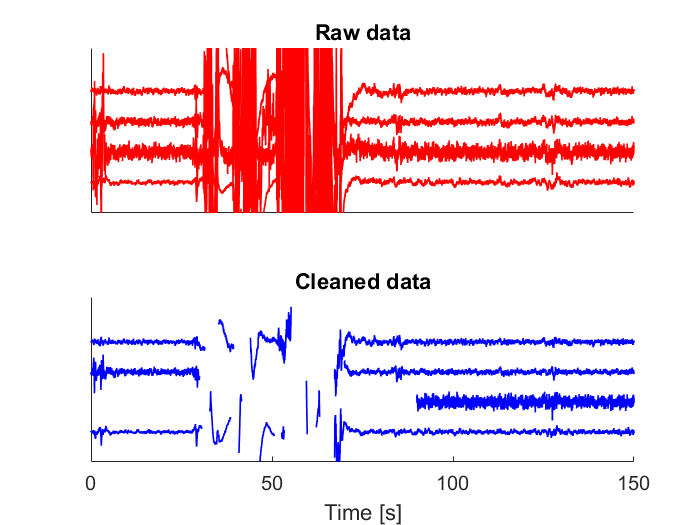

Supplement: Supplementary file 1 [file Data_Sheet_1.docx]
